# Prehospital prognosis is difficult in patients with acute exacerbation of chronic obstructive pulmonary disease

**DOI:** 10.1186/s13049-017-0451-4

**Published:** 2017-11-02

**Authors:** Katrine P. Lindvig, Anne C. Brøchner, Annmarie T. Lassen, Søren Mikkelsen

**Affiliations:** 1 Mobile Emergency Care Unit, Department of Anaesthesiology and Intensive Care, Odense University Hospital, Junggreensvej 8, 1. tv, 5000 Odense C, Region of Funen Denmark; 2 Institute of Clinical Research, University of Southern Denmark, Odense, Denmark; 3 Department of Emergency Medicine, Odense University Hospital, Odense, Denmark

**Keywords:** Chronic obstructive pulmonary disease, Prehospital, Emergency, Intensive care, Mortality, Prognostic

## Abstract

**Background:**

Patients with acute exacerbation of chronic obstructive pulmonary disease often require prehospital emergency treatment. This enables patients who are less ill to be treated on-site and to avoid hospital admission, while severely ill patients can receive immediate ventilatory support in the form of intubation. The emergency physician faces difficult treatment decisions, however, and prognostic tools that could assist in determining which patients would benefit from intubation and ventilator support would be helpful. The aim of the current study was to identify prehospital clinical variables associated with mortality from acute exacerbation of chronic obstructive pulmonary disease. As part of the study, we estimated the 30-day mortality for patients with this prehospital diagnosis.

**Methods:**

A retrospective study was performed using data collected by the mobile emergency care unit in Odense, Denmark, combined with data from the patients’ medical records. Patients with the tentative diagnosis of acute exacerbation of chronic obstructive pulmonary disease between 1st July 2011 and 31st December 2013 were included in the study.

**Results:**

Based on data from 530 patients, we found no statistically significant associations between prehospital clinical variables and mortality, apart from a minor association between older age and higher mortality. The overall 30-day mortality was 10%, while that for patients admitted to the intensive care unit was 30%.

**Conclusion:**

No specific prehospital prognostic factors for mortality were identified. Prognostic assessment and the decision to withhold treatment for acute exacerbation of chronic obstructive pulmonary disease seem inadvisable in the prehospital setting.

## Background

Chronic obstructive pulmonary disease (COPD) is an important cause of morbidity and mortality [1]. It has been estimated that approximately 8% of the Danish population has COPD [2], and many of these experience acute exacerbations (AE) mainly caused by respiratory infections. The resulting hypoxemia and hypercapnia can be severe and may require acute emergency care. The long-term consequences of acute exacerbations of COPD include reduced quality of life, increased risk of mortality, prolonged hospitalisation, and increased financial costs to the healthcare system [3–6].

Greater focus on prehospital treatment in the region of Southern Denmark led to the implementation in 2006 of mobile emergency care units (MECUs) staffed by physicians specialised in anaesthesiology. The use of specialist staff enables less ill patients to receive on-site treatment and avoid hospital admission [7], while critically ill patients with respiratory failure may also benefit from early physician-directed treatment [8]. It is therefore assumed that the MECU is a rational supplementary treatment for the large group of patients with acute exacerbations of COPD [5, 9].

The management of patients with acute exacerbations of COPD is complex, however. The urgency of the condition means that the MECU physician has little information and time at hand (on average 15 min at the scene). The physician has to quickly decide whether or not to initiate invasive procedures such as intubation and ventilator therapy, based on the immediate objective findings and without access to the patient’s medical files and information on the patient’s habitual condition, and with no possibility of collegial sparring and advice. The MECU physician must thus consider whether the potential risks of treatment outweigh the potential benefits for the patient [10].

The task of the MECU physician could be made easier if there was some way of determining the most likely prognosis for acutely ill patients with COPD in the prehospital setting. The physician would then have a better idea of which patients would be most likely to benefit from invasive treatment. The aim of this study was to identify clinical variables associated with mortality from acute exacerbations of COPD that could be used as prognostic indicators by the MECU physician. As part of the study, we estimated overall mortality for patients treated by the MECU for acute exacerbations of COPD, as well as 30-day mortality for patients admitted to the intensive care unit.

## Methods

We conducted a retrospective cohort study of adult patients (age ≥ 18 years) with acute exacerbation of COPD who were treated by the MECU based in Odense, Denmark, between 1st July 2011 and 31st December 2013. Patients were identified through the MECU registry, which covers all patients treated by the MECU. Patients were followed up until release at the scene, hospital discharge, or death. In line with the Utstein criteria, patient mortality was estimated for 30 days after MECU treatment. Data are reported according to the STROBE guidelines [11].

### The mobile emergency care unit

Throughout the study period, the Odense MECU was part of a two-tier system, alongside a standard ambulance manned with two emergency medical technicians (EMTs). The Odense MECU is a rapid-response car that operates all year round and is manned with a specialist in anaesthesiology and an EMT. The MECU covers an area of approximately 2500 km^2^ and serves a population of approximately 260,000 people. It is dispatched either by the emergency dispatch centre or at the request of the EMTs in the standard ambulance [12]. The MECU is called on average 4900 times per year, with an average of 13.5 calls per day. Due to overtriage, the standard ambulance cancels 20% of the calls while the MECU is en route. Approximately 11% of the patients seen by the MECU are released from care after on-site treatment [7].

Patients seen by the MECU comprise three major groups. Approximately 60% receive an observational diagnosis for symptoms, signs and/or abnormal clinical and laboratory findings that are not elsewhere classified or an injury diagnosis (ICD-10 groups R, S, T and Z) [13]. Approximately 19% of the patients are diagnosed with circulatory system diseases, and 9% are diagnosed with diseases of the respiratory system. Following each MECU run, the patient’s characteristics, vital parameters, diagnosis and outcome (released on-site, admitted to hospital, or declared dead on-site) are entered into the MECU database and the patient’s medical record. Due to the Danish Civil Registration System, each Danish resident has a unique civil personal register number for identification purposes [14]. Patients requiring hospital admission were taken to Odense University Hospital, which is an 1100-bed, level 1 trauma centre with all medical specialities.

### Data collection

Patients eligible for the study were those tentatively diagnosed by the MECU physician as having acute exacerbation of COPD (ICD-10 classification code J44.1) [13]. Patient data on gender, age, vital parameters (respiratory frequency measured manually and arterial oxygen saturation measured by pulse oximetry), respiratory status, and prehospital medication were extracted from the MECU database. The patient’s respiratory status was subjectively assessed by the prehospital physician according to four groups: unaffected, affected, severely affected, and manifest respiratory failure. This arbitrary assessment is not based on predefined scores, and one must expect that “unaffected” and “respiratory failure” have the highest consistency.

Data on in-hospital treatment and final diagnosis were extracted from the patient’s medical records. We used the in-hospital diagnosis to confirm the validity of the prehospital diagnosis of acute exacerbation of COPD. We did not collect spirometry data, either previous or related to the current episode, as all included patients were assumed to have acute deterioration of COPD. Completeness of data was ensured by two of the authors (KPL and ACB) individually checking each patient’s records manually.

### Statistical analysis

Patient baseline characteristics were presented as means and ranges for normally distributed variables, and as medians or percentages for skewed distributions. Univariate analyses were carried out and tested using the χ2 -test, which was two-sided. The association with death of predefined variables was evaluated in a univariate logistic regression analysis using the log-rank test with the variables included: age, gender, respiratory status, respiratory frequency, saturation, and medication (Salbutamol/Ipratropiumbromide and steroids). The 30-day mortality was reported as the proportion of patients who died within 30 days and was shown in a Kaplan–Meier failure plot. Furthermore, we performed two multivariate analyses, chosen per protocol. One: A multivariate logistic regression analysis with the purpose to identify risk factors for ICU admission and longer hospitalisation (>4 days). Two: A multivariate Cox regression analysis, with the purpose to identify prognostic factors associated with death for patients with AECOPD treated by the MECU physician. Variables included in the multivariable analysis, were those that were statistically significant (*p* value <0.05) in the univariate analysis, being age and gender. Patients who were included in the models were all included patients in the study, independently of hospital admission, or confirmation of AECOPD with reference to the aim of the study, being the prehospital point-of-view. The proportional hazard assumptions were tested graphically using the Cox–Snell residuals and found appropriate. The 95% confidence intervals were calculated based on a normal distribution of the estimates. Statistical tests were two-sided and a *p*-value of <0.05 was considered statistically significant. In the case of missing data, the values were registered as normal. Statistical analyses were performed using Stata 13.0 (Stata Corp LP, College Station, Texas).

### Ethics

The study was approved by the Danish Data Protection Agency (J.no. 2014–41-3212) and the Danish Health and Medicines Authority (J.no. 3–3013-1052/1/). According to Danish law, observational studies do not require patient consent or review by an ethics committee.

## Results

Within the study period of 2 years and 6 months, 537 patients were diagnosed with acute exacerbation of COPD by the MECU physician and were thus eligible for inclusion. Seven patients were lost to follow-up and were excluded from the study, giving 530 patients in the study. Patients could have had multiple episodes of acute COPD exacerbation, thus 278 patients had one contact with the MECU, and one patient had 15 contacts within the study period. The median age was 71.6 years (range: 43–100), and approximately half the patients had severely affected breathing (Table 1). The majority of patients were treated with salbutamol with or without steroids (Table 2).

**Table 1:** Baseline characteristics of patients with acute exacerbation (AE) of chronic obstructive pulmonary disease

Patient characteristics	Patients with AE *n* = 530 (range / %)
Sex	
Male	246 (46.4)
Female	284 (53.6)
Age (years), mean (range)	71.6 (43–100)
Respiratory frequency (breaths/min), mean (range)	26 (0–62)
First measured oxygen saturation (%)	87 (50–100)
Last measured oxygen saturation (%)	91 (50–100)
Respiratory status	
Unaffected	21 (3.9)
Affected	223 (42.1)
Severely affected	274 (51.7)
Respiratory failure	6 (1.1)

**Table 2:** Prehospital treatment of patients with acute exacerbation of chronic obstructive pulmonary disease (COPD)

Treatment	Patients with COPD *n* = 530 (%)
Prehospital setting	
Salbutamol/Ipratropiumbromide^a^	371 (70.0)
Salbutamol	183 (34.5)
Terbutaline	38 (7.1)
Steroids^b^	304 (57.3)
Furosemide	77 (14.5)
Nitroglycerine	29 (5.5)
Morphine	17 (3.2)
Intubation	8 (1.5)

^a^Only administered by prehospital physicians in Denmark

^b^Steroids administered often as intravenous treatment e.g. 125 mg methylprednisolone

### Hospitalisation

Of the 530 patients treated by the MECU, 61 (11.5%) were treated and released on-site by the MECU physician. This decision is usually made in cooperation with the patient and takes into account clinical parameters such as respiratory frequency, arterial oxygen saturation, and the general condition of the patient. None of the patients who were released at the scene by the MECU had any contact with the hospital-based health care system within 48 h of their MECU contact.

**Fig. 1:**
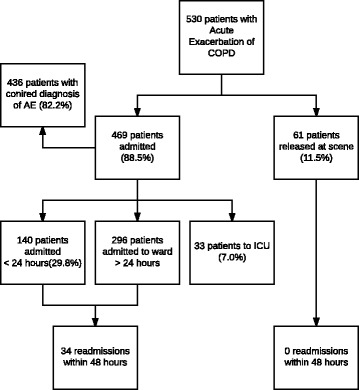
Flowchart of hospitalisation of included patients. ICU: Intensive Care Unit

The 469 (88.5%) patients who were admitted to hospital (Fig. 1) had a mean length of stay of 4.5 days (range: 0–48 days). At discharge, the diagnosis of acute exacerbation of COPD was confirmed in 436 (92.9%) of the 469 patients. The remaining 7% had a range of diagnoses including heart failure, pulmonary embolism, pneumothorax, and pneumonia.

Due to established hospital visitation guidelines, hospital patients are initially admitted to the emergency department for up to 48 h. We found that one-third (*n* = 140, 29.8%) of the hospitalised patients were discharged from the emergency department within 24 h of admission, and of these 13 (9.3%) were re-admitted within 48 h. Of the patients admitted to the department of respiratory medicine, 7.2% were re-admitted within 48 h of discharge.

Of the 469 patients admitted to hospital, 8.9% were admitted from their own home but were discharged to a nursing home, reflecting a substantial loss of daily function (Table 3).

**Table 3:** Outcome among patients with acute exacerbation of chronic obstructive pulmonary disease

Outcome	Patients with AE *n* = 530 (%)
Hospitalisation	
Released at the scene	61 (11.5)
Hospital admission	469 (88.5)
Discharged within 24 h	140 (29.8)
Discharged to home	387 (82.5)
Discharged to nursery home	42 (8.9)
Readmission within 48 h	34 (7.2)
Length of stay (days), mean (range)	4.5 (0–48)
Intensive Care Unit	*n* = 33 (6.2)
Intensive Care Unit admission	33
Total hours in ICU, mean (range)	51.1 (1–264)
Non-invasive ventilation^b^	85 (16.0 of all patients)
Invasive mechanical ventilation^a^	20 (60.6)
Prehospital intubation	8
Intubation within 3 h of admission	7
ICU 30-day mortality	10 (30.3)
Mortality	
0–30 day mortality	53 (10.0)
24-h mortality	14 (2.6)
30-day mortality	39 (7.4)

Overall mortality was sum of 24-h mortality and 30-day mortality

^a^Eight patients were intubated at prehospital scene, seven patients were intubated within 3 h of admission, four patients were intubated during ICU stay, and one intubated patient was lost to follow-up due to transfer to another hospital

^b^85 patients had non-invasive ventilation in the emergency department, ICU, or department of respiratory medicine

### Intensive care unit

Eight patients required prehospital intubation and were admitted to the intensive care unit (ICU). In total, 33/530 (6.2%) of patients were admitted to the ICU for an average of 2.1 days (51.1 h) and hospitalised in a total of 9 days (Table 3). Of these ICU patients, 31/33 (93.9%) were mechanically ventilated, on average for 25.2 h. A total of 85 patients received non-invasive ventilation, on average for 12.5 h (Table 3).

### Mortality and associated factors

**Fig. 2:**
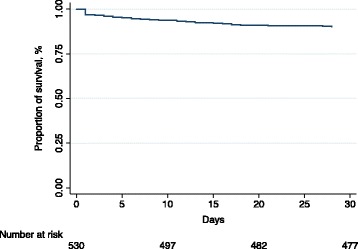
Kaplan-Meier survival estimate of patients with acute exacerbation of COPD

No patients were declared dead on-site. Fifty-three of the 530 (10%) patients died within 30 days of admission, including 14 patients (2.6%) who died within 24 h. Ten of the 33 (30.3%) patients treated in ICU died within 30 days of admission (Table 3) (Fig. 2).

The univariate analysis showed older age and male gender to be associated with higher mortality. Respiratory frequency, oxygen saturation, and respiratory status were not statistically significant in the univariate analysis. The multivariate logistic regression showed that male gender was associated with ICU admission, with an odds ratio of 2.9 (95%-CI 1.2–6.5), and that female gender was associated with shorter hospital stay, with an odds ratio of 0.5 (95%-CI 0.3–0.7). The multivariate Cox regression showed older age to be associated with mortality, with a hazard ratio of 1.04 (95% CI 1.01–1.06) per year, increasing from the age of 75 years.

## Discussion

Apart from an expected minor association between older age and higher mortality, we did not identify any prehospital prognostic indicators that could help the mobile emergency care physician to decide on treatment for acute exacerbations of COPD. Overall 30-day mortality for the patients seen by the emergency care unit was 10%, where 2.6% died in the first 24 h.

Although the rate of hospitalisation due to acute exacerbation of COPD has decreased in recent years, patients who are hospitalised have more severe disease in terms of higher mortality, comorbidity, and need for intensive care therapy [15, 16]. Despite this, we found that one-third of patients admitted with acute exacerbation of COPD were discharged from the emergency department within 24 h of admission, and only a small proportion of these patients were re-admitted. This probably reflects the wide heterogeneity of this patient group [17] and also the unpredictable course of the condition, where some patients quickly deteriorate while others improve within a relatively short period of time. Due to the experienced MECU anaesthesiologist’s awareness of the potential consequences for patients with respiratory difficulties, hospital admission for observation appears warranted, also for uncertain cases. We found that the prehospital MECU diagnosis of acute exacerbation of COPD was confirmed by the hospital discharge diagnosis in 90% of cases.

The mean length of hospital stay of 4.5 days (0–48) in our study is in line with other studies that report mean lengths of 5–12 days [4, 18–26]. In contrast to other studies [27, 28] however, our patients had a relatively short ICU stay (mean 2.1 days). This might be due to a high patient flow at the ICU that necessitates transfer to a medical ward as soon as the patient’s condition allows it. Another explanation may be the availability of non-invasive mechanical ventilation at other departments, rendering ICU admission unnecessary.

In contrast to the large group of less severely ill patients, our study included eight patients who were severely ill and required prehospital endotracheal intubation. Overall, 20% of our patients received mechanical ventilation during admission, of whom one-quarter received invasive mechanical ventilation. The general perception that the outcome for COPD patients requiring mechanical ventilation is poor may work against the choice to intubate and admit a patient to the ICU. Clinicians are often pessimistic about the prognosis for patients with COPD admitted to ICU [29, 30], and decisions regarding intubation and survival predictions can vary for identical COPD patients [31]. In our study, six patients were intubated immediately or within two hours of admission, and it may be argued that these patients should have been intubated at the prehospital scene. This apparent prehospital withholding of treatment may reflect the MECU physician’s pessimism regarding long-term survival of these patients, although hospitals are developing a more liberal approach to invasive mechanical ventilation due to improved ventilation techniques and greater knowledge about the conditions for successfully weaning patients off ventilators [32].

The mortality rates in our study are similar to those reported in other studies, with in-hospital mortality ranging from 4 to 30% [4, 33, 34] and a markedly higher mortality for patients requiring mechanical ventilation in the ICU [34]. The mortality rate for ICU patients in our study was lower than that of other ICU patient groups [35], however, possibly due to some of our ICU patients being less severely affected and receiving non-invasive mechanical ventilation due to lack of capacity in the general ward. These patients may not have fulfilled the usual criteria for ICU admission and thus had better outcome than the average ICU patient.

In accordance with other chronic diseases affecting the adult population, we found increasing age to be a significant prognostic factor for mortality. Gender, respiratory frequency, and oxygen saturation were not significantly associated with 30-day mortality. However, we found that the male gender was a significant predictor of admission to the ICU, while female gender was a predictor of shorter hospital stay. Previous studies have sought to identify in-hospital prognostic factors for COPD patients, but no clear picture has emerged. Although various disease-specific prognostic models have been developed, the use of such models is controversial as it supports estimates for patient groups and not for individual patients [6]. Prognostic models for COPD tend to perform poorly for patients admitted to hospital and seem less relevant for the prehospital setting due to the brief time spent on-site and the limited access to the patient’s previous medical data. In the present study, the prehospital examinations registered did not include spirometry. However, spirometry might have provided the MECU physician with indications that a given patient was entering the terminal phase of his disease, thus potentially influencing treatment. In the future, the prehospital personnel may have the potential to electronically access the patients´ in-hospital medical records. This will give rise to a substantially increased amount of knowledge concerning the patients´ habitual state, as well as the patients´ wishes regarding the level of treatment. Thus, it is highly possible that the treatment of some terminally ill patients with acute exacerbations of COPD in the future may be withheld prehospitally. However, based on our investigation, that time has yet to come.

One of the strengths of this study is the complete patient follow-up, due largely to the extensive registration practice in the Danish health care system based on individual identification numbers [14] and enabling us to follow patients from the prehospital setting, through the intensive care unit, and to discharge from hospital.

A major limitation in our attempt to develop prognostic tools was the relatively small sample size (the number of patients dying within 30 days from the index date). One might argue that the inclusion period could be extended further back in time. In recent years, however, the treatment possibilities have expanded within the prehospital medical services [36, 37], alongside improved diagnostic capabilities through point of care ultrasound and arterial blood gas analysis [38]. Therefore, the authors believe that the possible adjustments to create a larger sample size would result in a more heterogeneous patient population. Our study was also limited by being a single-centre study. However, Krüger et al. [39] have shown that the prehospital organisations in Scandinavia are generally comparable, and we believe that our results are generalisable to similar patient groups elsewhere in Scandinavia.

## Conclusion

This study showed that patients with acute exacerbation of chronic obstructive pulmonary disease are a heterogeneous group, where 10% could be released from care after on-site treatment and 30% of those admitted to hospital were discharged within 24 h, but 6% required mechanical ventilation in the intensive care unit with 30% mortality within 30 days. No prognostic factors predicting death could be routinely collected at the prehospital phase. Thus, we could not identify any specific prognostic factors for mortality that could help the mobile emergency care physician to decide on treatment options. Prognostic assessments and the decision to withhold treatment for acute exacerbation of chronic obstructive pulmonary disease seem inadvisable in the prehospital setting.

## Abbreviations

CI: Confidence interval
COPD: Chronic obstructive pulmonary disease
ICU: Intensive care unit
MECU: Mobile emergency care unit
